# Is blended learning and problem-based learning course design suited to develop future public health leaders? An explorative European study

**DOI:** 10.1186/s40985-018-0090-y

**Published:** 2018-06-01

**Authors:** Karen D. Könings, Nynke de Jong, Christa Lohrmann, Linas Sumskas, Tony Smith, Stephen J. O’Connor, Ingrid A. E. Spanjers, Jeroen J. G. Van Merriënboer, Katarzyna Czabanowska

**Affiliations:** 1 Department of Educational Development and Research and Graduate School of Health Professions Education, Faculty of Health, Medicine and Life Sciences, Maastricht University, PO Box 616, 6200 MD Maastricht, the Netherlands; 2 Department of Nursing Science, Medical University of Graz, Universitaetsplatz 4, A-8010 Graz, Austria; 3 Department of Preventive Medicine and Institute of Health Research at the Faculty of Public Health, Lithuanian University of Health Sciences, A.Mickeviciaus street 9, 44307 Kaunas, Lithuania; 4 Centre for Leadership in Health and Social Care, Faculty of Health and Wellbeing, Sheffield Hallam University, Sheffield, S10 2BP UK; 5 England Centre for Practice Development, Faculty of Health and Wellbeing, Canterbury Christ Church University, Canterbury, UK; 6 Department of International Health, CAPHRI School for Public Health and Primary Care, Faculty of Health, Medicine and Life Sciences, Maastricht University, PO Box 616, 6200 MD Maastricht, the Netherlands; 7 Institute of Public Health, Faculty of Health Sciences, Medical College, Jagiellonian University, Krakow, Poland

**Keywords:** Leadership, Public health, Problem-based learning, Blended learning, Competency self-assessment, Evaluation

## Abstract

**Background:**

Public health leaders are confronted with complex problems, and developing effective leadership competencies is essential. The teaching of leadership is still not common in public health training programs around the world. A reconceptualization of professional training is needed and can benefit from innovative educational approaches. Our aim was to explore learners’ perceptions of the effectiveness and appeal of a public health leadership course using problem-based, blended learning methods that used virtual learning environment technologies.

**Case presentation:**

In this cross-sectional evaluative study, the Self-Assessment Instrument of Competencies for Public Health Leaders was administered before and after an online, blended-learning, problem-based (PBL) leadership course. An evaluation questionnaire was also used to measure perceptions of blended learning, problem-based learning, and tutor functioning among 19 public health professionals from The Netherlands (*n* = 8), Lithuania (*n* = 5), and Austria (*n* = 6).

Participants showed overall satisfaction and knowledge gains related to public health leadership competencies in six of eight measured areas, especially Political Leadership and Systems Thinking. Some perceptions of blended learning and PBL varied between the institutions. This might have been caused by lack of experience of the educational approaches, differing professional backgrounds, inexperience of communicating in the online setting, and different expectations towards the course.

**Conclusions:**

Blended, problem-based learning might be an effective way to develop leadership competencies among public health professionals in international and interdisciplinary context.

## Background

Public health leaders in many countries are faced with the challenges like the aging populations with chronic disease and major issues regarding emerging or re-emerging infectious diseases, including uncontrolled epidemics, distrust towards vaccination, or antibiotic resistance. There is little doubt that effective public health leadership is essential given the significant financial pressures on health services and the need to deliver more with less resources [1]. However, few educational courses deliver the necessary competences and specific training in leadership [2–4]. A recent debate on public health leadership featured in *The Lancet* pointed out that leadership is still not common in most public health training programs at undergraduate, postgraduate, and continuing professional development level and asserted that every public health organization should be engaged in developing more leaders at every level [4, 5]. There is a need for substantial investment in leadership training for public health professionals [6]. This raises a question of how higher education institutions can provide “content and context to initiate a major reconsideration of working and learning patterns which incorporate novel forms, based on the principles of interprofessional collaboration and transcend the confines of the classroom” [7].

Blended learning, which is a combination of face-to-face and online learning [8], improves access to education for learners who need to organize their education around professional roles or domestic responsibilities or live far from universities [8–11]. Further, it is possible to provide the same educational program to learners in different countries [11, 12] and enable learning in groups despite geographical boundaries [13], which is important in the context of globalization in higher education and continuing education [14, 15]. This should facilitate multidisciplinary learning and nurture a spirit of teamwork, particularly with regard to leadership skills [16]. Blended learning has proven to be at least as effective and equally satisfying for learners as traditional learning methods [17–19].

Problem-based learning (PBL) [20] is widely used in medical education and stimulates the development of leadership competencies as learners are self-directed and collaborate in small groups to work on authentic, complex tasks to explore problems and consider possible solutions [21, 22]. In response to the need to develop effective public health leaders, we explored the perceptions of the effectiveness and appeal of a newly developed public health leadership course using problem-based, blended-learning methods using virtual learning environment technologies. It was developed by international experts in public health and implemented in an international context. As diversity between learners from different countries may influence the adaptation and use of educational innovations [23], we also investigated possible differences between three different European academic settings: the Netherlands, Austria, and Lithuania.

## Case presentation

This cross-sectional, evaluative study was carried out in two phases. At the beginning of the course, participants from all three countries filled out the Self-Assessment Instrument of Competencies for Public Health Leaders (SAIC-PHL) [24]. At the end, they again filled out an evaluation questionnaire and the SAIC-PHL.

### Course description

The Leadership in Public Health course^1^ was designed by international experts to introduce diverse European perspectives of leadership in the modern public health environment in Europe. Public health is broadly made up of a number of specific disciplines such as methods in public health; population health and its social and economic determinants; population health and its material-physical, radiological, chemical, and biological environmental determinants; health policy; economics; organizational theory and management; health promotion; health education; health protection; and disease prevention and ethics [25]. A starting point was to conceptualize public health in a way that it was relevant to all European states. For this reason, the project adopted the definition of public health in line with the European Public Health Operations (EPHOs) which constitute, “a set of fundamental actions that address determinants of health, and maintain and protect population health through organized efforts of society [26].” It was built around a thematic framework of public health leadership competencies, based on a systematic literature review [27], consisting of 52 competencies distributed among eight domains: Systems Thinking, Political Leadership, Building and Leading Interdisciplinary Teams, Leadership and Communication, Leading Change, Emotional Intelligence and Leadership in Team-based Organisations, Leadership Organisational Learning and Development and Ethics and Professionalism. After being first piloted by Sheffield University in the UK, the course was implemented at Maastricht University (Netherlands), Kaunas University (Lithuania), and the University of Graz (Austria) which were partners in the EU Erasmus Curriculum Development project “Leaders for Public health in Europe.” It was a part-time course, delivered over a period of 8 weeks. The official language was English. PBL was used as the instructional model and implemented as blended learning. The course began with one and a half days of face-to-face learning which included introductions to blended learning and PBL, tutorial group meetings for the first and second PBL task, and a lecture. All other tutorial group meetings and lectures were delivered online during six half-day sessions within a period of 8 weeks. The online sessions were interactive. Participants could interact by using the microphone or the chat function.

Collaboration is one of the key learning principles of PBL and played a central role during the tutorial group meetings. During these meetings, participants were continuously in interaction with each other. Knowledge and experiences were shared, like in proper face-to-face PBL meetings. All participants attended the online lectures as one group. In some lectures, small group events were organized: Participants were divided into different online breakout rooms for discussions and afterwards plenary reported the results of that discussion in the main online lecture hall.

The taught content was based on the competency domains and included systems thinking, political leadership, collaborative leadership, building and leading interdisciplinary teams, leadership and communication, leading change, emotional intelligence and leadership in teams, and leadership, organizational learning, and development. These topics correspond with domains and competencies associated with leadership in the public health domain and cover the topics that were identified in the literature as most important and relevant for public health leaders [27].

Each session was delivered by a teacher(s) responsible for one of these components. Teachers were from the UK, Austria, Lithuania, and the Netherlands and represented various academic fields: public health, psychology, nursing, political science, education, and social science. All teachers underwent PBL training and blended learning training prior to the delivery of the course. They also served as tutors in the online tutorial groups.

A virtual learning environment was constructed which contained announcements, course information including course handbook, information on e-learning, and information for the sessions including all teaching materials, such as assignments, briefs, hand-outs, additional references, and literature cited or used during each session. There was also a discussion board, which was used for informal communication between participants. E-mail was used for questions to the course coordinator.

### Participants

Nineteen participants (4 males, 15 females) completed the course: eight from Maastricht University (The Netherlands), five from Kaunas University (Lithuania), and six from the Medical University of Graz (Austria). The participating universities offered the existent courses in which this leadership course in a blended learning format could be included. The participants from Maastricht followed the European Public Health program and were used to PBL tutorial groups and familiar to some online practices. Participants from Kaunas followed a public health PhD program and had no prior experience of PBL or online learning, but they did have experience of team-based learning. Participants from Graz were working professionals, following a Master’s Program in Health and Nursing Science and were usually taught in lectures and small group seminars, but also had some experience of PBL as well as some experience with web-based-training and online lectures.

### Instruments

The Evaluation Questionnaire consisted of 54 items from several existing scales. General satisfaction with course and instructor quality was measured with three items [28]. Thirteen items were used for measuring instructiveness, productivity of tutorial groups, applicability of new knowledge, and difficulty of the course. Tutorial group functioning was measured by six subscales: elaboration, interaction, motivation, and sponging, cohesion, and withdrawing [29]. To evaluate tutor functioning, four subscales were used [30]: stimulating constructive/active learning, stimulating self-directed learning, stimulating contextual learning, and stimulating collaborative learning. Motivation of the tutor to fulfill this role and stimulating professional behavior by the tutor are two single-item scales. Quality of different aspects of the e-learning was evaluated with three subscales [10]: Evaluation of e-teaching, evaluation of e-resources, and interactions between learners consisted. Items use a 5-point Likert scale ranging from strongly disagree to strongly agree or a 10-point rating scale. One additional item asked to rate their computer skills on a 5-point rating scale ranging from “very poor” to “excellent.”

The Self-Assessment Instrument of Competencies for Public Health Leaders (SAIC-PHL) [27] was used to measure learners’ own perceptions of their competencies before and after the course. This consisted of 52 items describing competencies essential for public health leaders. These competency descriptions are developed based on a literature review and refined and validated in a consensus development panel and two rounds of a Delphi survey [27]. For each competency, learners had to assess how well they thought they were doing on a 5-point scale ranging from “acting as a novice” to “acting as an expert.” This scale has been adapted from Dreyfus and Dreyfus [31]. The items were organized into eight competency domains of the public health leadership framework [27], reflected in the eight subscales of the SAIC-PHL.

### Data collection and analyses

All data were collected online. SPSS version 19 was used to analyze the data. Cronbach’s alpha was calculated for the different scales from the Evaluation Questionnaire and the SAIC-PHL to check whether it was acceptable to use scale scores (alpha from .70 considered as acceptable, from .80 as good). We also explored the results on scales with lower alpha scores, while interpreting the results with more caution. Descriptive statistics for items and scales of the Evaluation Questionnaire, in the form of percentages and means, were used to examine the participants’ evaluations of the course. For negatively formulated items and scales consisting of only negatively formulated items, smaller means were interpreted as more positive. For scales containing both negatively and positively formulated items, the negatively formulated items were recoded before scale scores were calculated. For the SAIC-PHL, pretest and posttest scores were compared with paired *t* tests.

Differences in evaluation scores and in mean gains on the SAIC-PHL between participants from different locations were analyzed with non-parametric Kruskal-Wallis tests and post hoc Mann-Whitney tests. Non-parametric tests were used, because the sample sizes were small due to splitting up the sample into three groups for these analyses. To correct for multiple testing, Bonferroni-corrected alpha levels were used with the paired *t* tests, Kruskal-Wallis tests, and the Mann-Whitney tests. For the Kruskal-Wallis tests, Bonferroni correction was applied for each instrument separately. For the Mann-Whitney tests, the correction was applied for each pair of post hoc tests after a significant Kruskal-Wallis test. Bonferroni correction was also applied for the eight paired *t* tests computed for the SAIC-PHL.

## Results

The response rate for pretest and posttest was 100%. With regard to the Evaluation Questionnaire, the subscale for global rating of course and instructor quality showed good internal consistency (*α* = .89). Subscales on tutorial group functioning showed acceptable to good Cronbach’s alphas consistency for elaboration (*α* = .85), interaction (*α* = .76), motivation (*α* = .93), and sponging (*α* = .73). Because for cohesion and withdrawing found alphas were below .50, scores on separate items were used in the analyses. Concerning tutor functioning, the results were the following: stimulating constructive/active learning (*α* = .85), stimulating self-directed learning (*α* = .58), stimulating contextual learning (*α* = .81), and stimulating collaborative learning (*α* = .89). With respect to e-learning, it showed evaluation of e-teaching (*α* = .90), evaluation of e-resources (*α* = .85), and interactions between learners consisted (*α* = .66). Table 1 presents Cronbach’s alphas of the SAIC-PHL.

**Table 1:** Cronbach’s alphas for the scales from the self-assessment of competencies for public health leaders on pretest and posttest

	*N* of items	*α* pretest	*α* posttest
Systems Thinking	7	.89	.91
Political Leadership	8	.94	.95
Inspiring and Motivating Others	7	.93	.96
Building and Leading Interdisciplinary Teams	5	.82	.95
Leadership and Effective Communication	7	.86	.89
Leading Change	6	.95	.94
Emotional Intelligence and Leadership in Teams	6	.91	.94
Ethics and Professionalism	6	.95	.93

### Evaluation of the course

#### General satisfaction

The course content was just right according to 15 of the participants (79%), while two of them (11%) thought contents were easy and two others (11%) found them difficult. The average grade given to different aspects of the course ranged from 6.79 (SD = 2.66) to 7.79 (SD = 2.04) on a scale from 1 to 10. Because these means are on the positive half of the scale, these results indicate that on average participants were satisfied. Scores for the items using a 5-point scale differed between 3.47 (SD = 1.07) and 3.95 (SD = 0.97); they were on the positive side of the neutral value of 3. Means for the two negatively formulated aspects were 1.74 (SD = 0.93) and 2.05 (SD = 1.35), indicating positive evaluations. On the scale “Global rating of course and instructor quality,” the score was 3.53 (SD = 1.04).

Table 2 shows results separately for differences between the locations. Due to the Bonferroni correction (accounting for conducting 14 tests), an alpha of .0036 is used for the Kruskal-Wallis tests. For the Mann-Whitney tests, the corrected alpha was .025. Participants from Graz (*M*_Graz_ = 4.56, SD = 0.66) gave higher global ratings of course and instructor quality than their peers (*M*_Maastricht_ = 3.33, SD = .82, *U* = 5.50, *p* = .01) (*M*_Kaunas_ = 2.60, SD = 0.64, *U* = 1.00, *p* = .0087).

**Table 2:** Student evaluation of course on leadership: results of Kruskal-Wallis comparing results at different locations

Evaluation items	*H*	*p*
The organization of the block^a^	3.64	.16
The instructiveness of the block^a^	2.43	.31
The link of the block with their prior knowledge^a^	0.79	.69
The productivity of the tutorial group^a^	9.09	.0052
The link between the block and the assessment^a^	3.74	.16
The objectives of the course were clear^b^	4.74	.08
The literature fitted to the objectives of the course^b^	6.55	.03
I can apply what I have learned in my daily work^b^	2.88	.23
The e-lectures were instructive^b^	7.35	.02
The tasks in the course handbook were instructive^b^	6.27	.04
There were problems in collaboration because of differences in cultural background^b^	5.28	.07
There were difficulties because different universities were involved^b^	5.18	.07
Difficulty of the block contents^b^	1.46	.62
Scale “global rating of course and instructor quality”^b^	9.71	.0031

^a^Grades given on a 10-point scale

^b^5-point Likert scale

#### Group functioning

The means on functioning of the tutorial group were 3.56 (SD = 0.79) for interaction, 3.58 (SD = 1.07) for motivation, and 3.87 (SD = 0.78) for elaboration. Also, on cohesion, the means (3.11, SD = 1.37 and 4.26, SD = 0.65) were on the positive side of the scale or close to the neutral value of 3. For the scale on negative aspects of group functioning (i.e., sponging) the mean score was 2.58 (SD = 1.02); a lower score is a positive outcome on this scale. For the two items on withdrawing, the means were 3.58 (SD = 1.22) and 2.32 (SD = .82).

Table 3 presents the differences between locations. Due to the Bonferroni correction (accounting for conducting 8 tests), an alpha of .0063 is used for the Kruskal-Wallis tests of group functioning. Significant differences were found for the scales interaction and sponging and for one of the items on withdrawing. Participants from Graz were more positive than the others about interaction and about the absence of sponging and withdrawing.

**Table 3:** Evaluation of functioning of the tutorial group and tutor functioning: descriptive statistics and results of Kruskal-Wallis tests and Mann-Whitney tests for different locations

	*M* (SD) Maastricht	*M* (SD) Kaunas	*M* (SD) Graz	KW	MW Maastricht-Kaunas	MW Graz-Maastricht	MW Graz-Kaunas
Scales				*H (p)*	*U (p)*	*U (p)*	*U (p)*
Functioning of tutorial group							
Elaboration	3.44 (0.68)	3.70 (0.45)	4.58 (0.66)	6.61 (.03)			
Interaction	3.00 (0.59)	3.47 (0.38)	4.39 (0.53)	11.34 (.0005)	10.50 (.17)	1.00 (.0013)	2.00 (.02)
Motivation	3.31 (0.88)	2.90 (1.19)	4.50 (0.55)	7.67 (.01)			
Sponging	2.75 (0.76)	3.50 (0.71)	1.58 (0.66)	10.90 (.001)	9.50 (.13)	5.50 (.0097)	.00 (.0043)
Cohesion_item 1	4.38 (0.52)	3.80 (0.45)	4.50 (0.84)	3.94 (.14)			
Cohesion_item 2	3.25 (1.28)	3.40 (1.34)	2.67 (1.63)	0.86 (.68)			
Withdrawing_item 1	4.00 (1.07)	4.20 (0.84)	2.50 (1.05)	6.70 (.03)			
Withdrawing_item 2	2.63 (0.74)	2.80 (0.45)	1.50 (0.55)	9.31 (.0047)	18.50 (.84)	6.00 (.02)	1.50 (.0087)
Tutor functioning							
Stimulating constructive/active learning	3.38 (1.16)	3.47 (0.56)	4.50 (0.41)	7.50 (.02)			
Stimulating self-directed learning	3.13 (1.16)	3.40 (0.65)	3.92 (0.74)	1.86 (.41)			
Stimulating contextual learning	3.00 (0.80)	3.10 (0.82)	4.33 (0.52)	8.92 (.0064)	18.50 (.84)	4.00 (.0073)	2.00 (.01)
Stimulating collaborative learning	2.31 (1.16)	3.40 (0.65)	3.92 (0.80)	6.93 (.02)			
Motivation of the tutor	3.38 (0.74)	3.40 (0.55)	4.50 (0.84)	6.28 (.04)			
Stimulating professional behavior	2.63 (0.92)	3.20 (0.84)	4.33 (0.82)	8.28 (.00832)	13.00 (.36)	4.00 (.0087)	5.00 (.07)

#### Tutor functioning

The means for the tutor evaluation scales ranged from 3.11 (SD = 1.15) for stimulating collaborative learning to 3.75 (SD = 0.95) for stimulating constructive/active learning, indicating neutral to positive perceptions. The Bonferroni-corrected alpha for the Kruskal-Wallis tests of tutor functioning was .0083 (six tests conducted). Differences between locations were found on stimulating contextual learning and stimulating professional behavior (see Table 3). Participants from Graz were more positive about the tutors than participants from Maastricht on stimulating contextual learning and stimulating professional behavior. They were also more positive about stimulating contextual learning compared to participants from Kaunas.

#### Quality of blended learning

The mean regarding perceived quality of e-teaching was 3.61 (SD = 0.84), regarding quality of e-resources 3.49 (SD = .94), and regarding online student interaction 3.54 (SD = 0.71). The Bonferroni-corrected alpha for the Kruskal-Wallis tests of quality of blended learning was .017 (three tests conducted). Participants from Graz were more satisfied with the e-learning resources (*H* [2] = 9.23, *p* .0045; *M* = 4.33, SD = 0.63) than the participants from Kaunas (*M* = 2.60, SD = 0.86, *U* = 1.00, *p* = .01). On the other two scales, there were no differences.

#### Required computer skills

The average rating for own computer skills was 4.16 (SD = 0.90), ranging from poor to excellent. Because only one Kruskal-Wallis test was performed for required skills, alpha was not adjusted. Differences between the universities for computer skills were found, *H* [2] = 10.18, *p* = .003, indicating that participants from Graz (*M* = 3.17, SD = 0.75) rated their computer skills as poorer than those of the other participants (*M*_Maastricht_ = 4.63, SD = 0.52, *U* = 3.00, *p* = .005; *M*_Kaunas_ = 4.60, SD = 0.55, *U* = 2.00, *p* = .02). No differences were found between participants from Maastricht and Kaunas, *U* = 19.50, *p* = 1.00.

### Self-assessment of competencies for public health leaders

On the pretest, the SAIC-PHL scores on self-assessed competencies varied between 1.91 (SD = 0.83) and 3.02 (SD = 0.84). On the posttest, scores were between 2.89 (SD = 0.82) and 3.28 (SD = 0.74). The gains between pretest and posttest varied between 0.26 (SD = 0.96) and 1.04 (SD = 0.56). The Bonferroni-corrected alpha level for the parametric *t* tests analyzing the significance of the perceived learning gains and for the non-parametric Kruskal-Wallis tests analyzing the differences between the different locations was .0063. Significant learning gains were found for six of the eight competency domains: systems thinking, political leadership, inspiring and motivating others, building and leading interdisciplinary teams, leadership and effective communication, and leading change (see Table 4). Kruskal-Wallis tests showed that scores on different locations did not differ from each other.

**Table 4:** Self-assessment of competencies on leadership: descriptive statistics for pretest, posttest, and learning gains and statistics for paired *t* tests

Leadership domain (topic of teaching)	*M* (SD) pretest	*M* (SD) posttest	*M* (SD) gains	*t* (*df* = 18)	*p*
Systems Thinking	2.28 (0.65)	3.26 (0.59)	0.98 (0.69)	6.21	.0000
Political Leadership	1.91 (0.83)	2.95 (0.75)	1.04 (0.56)	8.10	.0000
Inspiring and Motivating Others	2.29 (0.74)	3.15 (0.81)	0.86 (0.76)	4.92	.0001
Building and Leading Interdisciplinary Teams	2.49 (0.57)	3.21 (0.84)	0.72 (0.53)	5.84	.0000
Leadership and Effective Communication	2.59 (0.63)	3.11 (0.73)	0.51 (0.59)	3.79	.0013
Leading Change	2.17 (0.92)	2.89 (0.82)	0.73 (0.83)	3.82	.0013
Emotional Intelligence and Leadership in Teams	3.02 (0.84)	3.28 (0.74)	0.26 (0.96)	1.20	.25
Ethics and Professionalism	2.81 (0.89)	3.10 (0.82)	0.29 (0.94)	1.35	.20

## Discussion and conclusions

The study showed that participants were overall positive about the effectiveness and delivery of the public health leadership course using blended learning and PBL methodologies. It seems that they valued both group functioning and tutor functioning in PBL as well as different aspects of blended learning (online interaction, e-teaching, e-resources). The self-reported level of leadership competencies increased over the period of the course for participants at all three locations. They gained most in the area of Political Leadership, Systems Thinking, and Inspiring and motivating others. The perceptions of blended learning and PBL partly varied between the participating locations. Differences in educational background between learners from different countries might have influence the use of educational innovations [23] as well as the perceptions of blended learning curricula [32, 33]. Keller and colleagues reported that in their study of blended education delivered in Lithuania, Sweden, and Norway, the students from Lithuania evaluated the virtual learning most positively, which is not confirmed in our study with participants from Kaunas University. We consider several possible explanations for the differences we found between evaluations of participants at different locations.

Lack of experience with PBL and online learning might have led to less positive evaluations of the course. Furthermore, participants’ expectations of the course might have differed from their experiences during the course due to perceived pressure and insecurity resulting from the need to perform in the group online. Discrepancies between expectations and actual experiences might have negatively affected their evaluation of the course [34]. The demanding nature of blended learning may negatively affect student reactions to these courses [17, 35], and lower activity of some participants in online discussions might be due to the fact that they were not used to actively participate in their learning at their home institution. Furthermore, the participants had different professional status. Participants who were clinical workers might have seen the relevance of the content more than participants who were mainly master students and PhD students. While the participants might have experienced the tension between the leadership theory and public health content, which constituted a kind of comfort zone for them, the resulting reflection contributed to a positive learning experience and understanding. In order to successfully implement effective public health interventions and change, collaboration with and participation of diverse groups of stakeholders is vital.

Although the results of the study can be direction setting for the blended PBL course designers, there are limitations to this study. The sample size of our study was small and selected conveniently using the available cohorts of students. The results could have been different if we offered the course on the open enrollment basis. It is a strength of our study that a pretest-posttest design was used for measuring competency gains, although based on self-assessment. Using objective learning data and standard assessment of participants’ competencies could be an interesting addition for future studies as well as following the impact of the course of a longer period of time and within an experimental design.

Taken together, this study suggests that organizing continuing education for professionals in an international context has potential [15]. Course designers might benefit from our results when designing blended learning courses. Bringing together international experts in the field as teachers in a blended course, with ample opportunities for group work and discussions among participants, contributes to the development of professional competencies, in our case on leadership in public health [13]. Differences between the evaluations of participants from universities from different countries point to the relevance of participants’ expectations, previous learning experiences, and educational context. The findings may also suggest that although the blended learning format of course delivery was well accepted in this small-scale study, there is a need to support students’ shift to the more active independent mode of learning including coaching, mentoring, and personal development planning. Such approaches are successfully included in other hybrid inline leadership courses, for example, University of North Carolina and Gillings School of Global Public Health Doctoral Program in Health Leadership [36]. The course applies modern technology and flexible teaching approaches with the emphasis on development of leadership competencies for experienced health professionals working full-time anywhere in the world. Similar to our course, leadership is learned through interaction, debate and collaboration, and mentorship which is a key to lifelong learning. Future research on best ways to support the learning process may also be relevant to the other academic environments. We recommend investing in training problem-based learning and blended learning skills for both students and staff to prevent that learning of these skills interferes with studying the course content.

## Conclusion

It seems that problem-based learning and blended learning can be an effective way to develop public health leadership competencies among professionals in international and interdisciplinary context if the specificity and educational background of the learners is properly addressed.

## Footnotes

1 The study “Leaders for European Public Health” (LEPHIE) Project was carried out with the support of the European Commission Lifelong Learning Program.
